# Clinical significance of left atrial geometry in dilated cardiomyopathy patients: A cardiovascular magnetic resonance study

**DOI:** 10.1002/clc.23529

**Published:** 2020-12-09

**Authors:** Mayu Yazaki, Takeru Nabeta, Takayuki Inomata, Kenji Maemura, Takumi Oki, Teppei Fujita, Yuki Ikeda, Shunsuke Ishii, Takashi Naruke, Yusuke Inoue, Junya Ako

**Affiliations:** ^1^ Department of Cardiovascular Medicine Kitasato University School of Medicine Sagamihara Kanagawa Japan; ^2^ Department of Cardiovascular Medicine Kitasato University Kitasato Institute Hospital Tokyo Japan; ^3^ Department of Diagnostic Radiology Kitasato University School of Medicine Sagamihara Kanagawa Japan

**Keywords:** cardiac magnetic resonance, dilated cardiomyopathy, heart failure, left atrial parameter

## Abstract

**Background:**

Clinical significance of left atrial (LA) function and geometry in patients with dilated cardiomyopathy (DCM) remains uncertain.

**Hypothesis:**

LA geometric parameters assessed by cardiac magnetic resonance (CMR) predict the prognosis in patients with DCM.

**Methods:**

The present study included patients with DCM and sinus rhythm who underwent CMR between December 2007 and April 2018. LA volume was measured using CMR. LA sphericity index was computed as the ratio of the measured maximum LA volume by the volume of a sphere with maximum LA length diameter.

**Results:**

We included 255 patients in this study. During the mean follow‐up of 3.92 years, hospitalization for HF occurred in 37 patients. The LA sphericity index was significantly higher in patients with hospitalization for HF than in those without (0.78 ± 0.35 vs. 0.58 ± 0.18, *p* < .001). Multivariable Cox regression analysis identified a higher LA sphericity index as an independent predictor of hospitalization for HF. Patients were categorized based on the median of LA sphericity index. The Kaplan–Meier curve showed that patients with a high LA sphericity index (≥0.57) had a significantly higher risk of hospitalization for HF than those with a low LA sphericity index (<0.57).

**Conclusion:**

LA sphericity index was an independent predictor of hospitalization for HF. Assessment of LA geometric parameters might be useful for risk stratification in patients with DCM.

## 1 INTRODUCTION

Dilated cardiomyopathy (DCM) is a myocardial disease typically diagnosed by impaired left ventricular (LV) ejection fraction and LV dilation.^1^ Guideline‐directed medical therapy (GDMT) improved the prognosis of patients with DCM.^2^ However, some patients with DCM still have poor outcomes.^3^, ^4^ It is important to reveal the factors that predict the prognosis to determine appropriate GDMT in patients with DCM.

The roles of the left atrium include modulating LV filling and cardiovascular performance by functioning as a reservoir for pulmonary venous return during ventricular systole, serving as a conduit for pulmonary venous return during early ventricular diastole, and providing a booster pump that augments ventricular filling during late ventricular diastole.^5^ Left atrial (LA) enlargement is associated with cardiac events in patients with LV diastolic dysfunction^6^ and atrial fibrillation.^7^ LA enlargement is also associated with cardiac events in patients with DCM.^8^ However, the clinical significance of LA function and geometry in patients with DCM remains uncertain.^9^ To our knowledge, although the previous study has found that the LV sphericity index was prognostic predictor in patients with DCM,^10^ there is no paper on the sphericity of the left atrium in patients with DCM. We believed that LA function and geometric change, especially spherical change, might be related to prognosis.

Cardiac magnetic resonance (CMR) is a suitable modality for investigating the properties, functions, and geometry. In addition, CMR is the current gold standard modality to assess LA function and geometry.^11^, ^12^ Thus, the aim of the present study was to investigate the relationship between the prognosis and LA parameters assessed by CMR in patients with DCM.

## 2 METHODS

### 2.1 Study subjects

This was a single‐center, retrospective, observational study of patients with DCM who underwent CMR at Kitasato University Hospital in Japan between December 2007 and April 2018. This study included both inpatients and outpatients. DCM patients were defined as LV ejection fraction <50% at CMR imaging. Patients with significant coronary artery disease (>75% luminal stenosis on coronary angiography or positive in nuclear cardiology), cardiac amyloidosis, sarcoidosis, acute myocarditis, metabolic disorders, endocrine dysfunction, neuromuscular diseases, peripartum cardiomyopathy, organic heart valve disease, the use of cardiotoxic drugs, and alcohol abuse were excluded. In addition, patients with atrial fibrillation and missing data were excluded. The Kitasato University Medical Ethics Organization has approved this study. The Organization approved this retrospective analysis of clinically acquired data and waived the need for written patient informed consent.

### 2.2 CMR image acquisition

CMR examinations were performed on a 1.5T (GE Healthcare, Milwaukee, WI, USA) or 3.0T (Siemens Healthineers, München, Germany) imaging scanner using a standard protocol. Cine images in short‐axis, long‐axis, and four‐chamber views were acquired using a breath‐hold cine steady‐state free precession sequence. Late gadolinium enhancement (LGE) images were acquired 10–15 minutes after the intravenous injection of 0.2 mmol/kg gadolinium using a segmented inversion recovery fast gradient‐echo sequence (1.5T) or true fast imaging with steady state free procession sequence (3.0T). Inversion times were optimized to null the myocardium.

### 2.3 CMR image analysis

LV and right ventricular volumetric analysis were performed offline with commercially available software (cvi42, version 4.1.8, Circle Cardiovascular Imaging, Calgary, Canada). The presence of LGE was defined as a liner mid‐wall in the septum or multiple pattern^13^ by the well‐trained operator.

**FIGURE 1:**
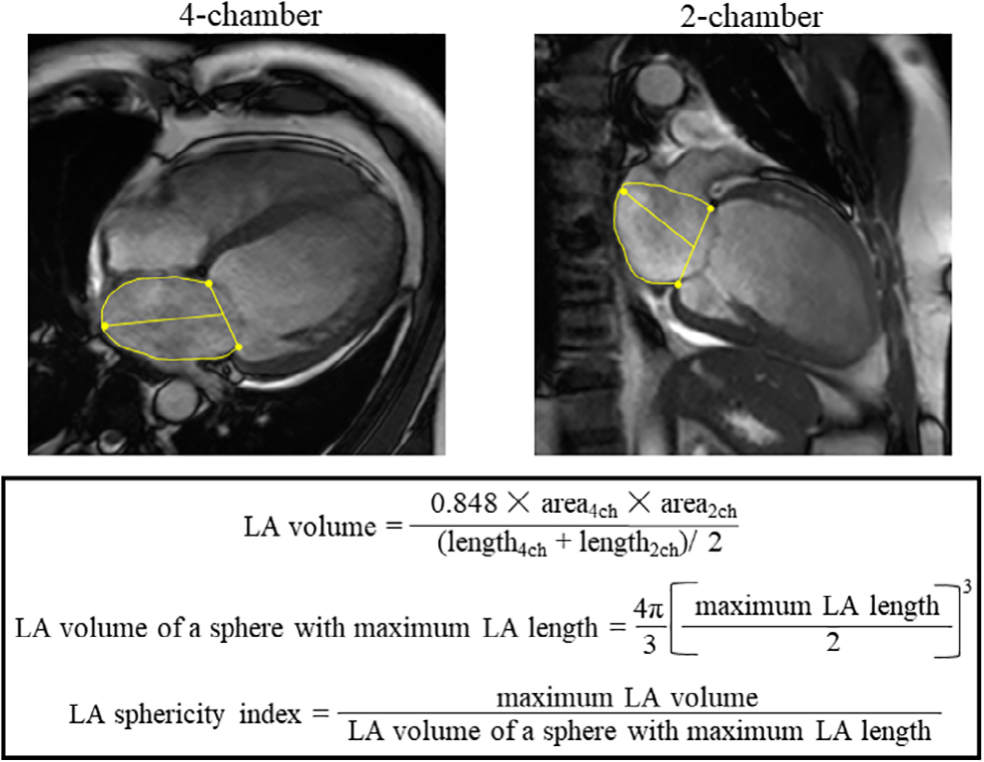
Measurement of LA volume and sphericity index. Left atrial (LA) volume was calculated from the 4‐ and 2‐chamber views. LA sphericity index was the ratio of the measured maximum LA volume to the volume of a sphere with maximum LA length diameter

LA volumes were assessed at three time‐points of the cardiac cycle: LA maximal volume at LV end‐systole before mitral valve opening (LAV_max_), LA volume at LV diastole immediately prior to LA contraction (LAV_pre−ac_) and LA minimal volume at late LV diastole after LA contraction (LAV_min_). Each phase was visually determined and the LA volumes were calculated from apical 4‐ and 2‐chamber views. In the 4‐chamber view, the LA border started from the medial side of the mitral annulus and included interatrial septum, posterior, and lateral LA walls and ended at the lateral mitral annulus. In the 2‐chamber view, the analyzed atrial border started from anterior mitral annulus and continued over the LA roof, the posterior wall, and the floor of the LA and ended at the inferior mitral annulus. LA length was the long‐axis length of LA from each chamber. The images of the 4‐ and 2‐chamber view are shown Figure 1. The LA volume was calculated using this formula: LA volume = (0.848 × area_4ch_ × area_2ch_)/([length_4ch_ + length_2ch_]/2).^14^ LA emptying fractions (LAEF) were calculated from the measured LA volumes as follows^14^, ^15^:

$$\text{Total LAEF}=\frac{\left({\text{LAV}}_{\max}\text{‐}{\text{LAV}}_{\min}\right)}{{\text{LAV}}_{\max}}\times 100$$

$$\text{Active LAEF}=\frac{\left({\text{LAV}}_{\text{pre‐ac}}\text{‐}{\text{LAV}}_{\min}\right)}{{\text{LAV}}_{\text{pre‐ac}}}\times 100$$

$$\text{Passive LAEF}=\frac{\left({\text{LAV}}_{\max}\text{‐}{\text{LAV}}_{\text{pre‐ac}}\right)}{{\text{LAV}}_{\max}}\times 100$$

The LA sphericity index was computed as the ratio of the maximum LA volume by the volume of a sphere with maximum LA length diameter (Figure 1).^16^

### 2.4 Clinical measurement and observation

As clinical evaluations at baseline, blood pressure, heart rate, laboratory examination, and echocardiography were measured upon undergoing CMR. Baseline characteristics and CMR were collected at the same time. The day when CMR was performed was set to zero. On echocardiography, LV volumes were calculated using the biplane method of disk summation (modified Simpson's rule), and the LV ejection fraction was measured as the difference between the end‐diastolic volume and end‐systolic volume divided by the end‐diastolic volume.^17^ Patients were treated according to GDMT, including beta‐blockers and renin‐aldosterone system inhibitors.^18^ Cardiac events were based on medical records. The primary endpoint was hospitalization for worsening heart failure (HF). Based on the time of CMR, we classified the study cohort into two groups by the presence or absence of subsequent hospitalization for HF. The hard endpoint was implantation ventricular assist device, death due to pump failure or lethal arrhythmia, or sudden cardiac death.

### 2.5 Statistical analysis

Data are presented as mean ± SD or as frequency (percentage). Student's *t*‐test was used to compare continuous variables, Wilcoxon‐Mann–Whitney test was used to evaluate the non‐normally distributed continuous variables, and Pearson's chi‐squared test was used to evaluate categorical variables. Two‐tailed *p* < .05 was considered to indicate statistical significance. Univariate Cox proportional hazard analysis was used to assess the association between each variable and cardiac event. Clinical variables with *p* < .05 as per univariate analysis were included for multivariate analysis. Event‐free survival curves were drawn according to the Kaplan–Meier method and compared using the log‐rank test. Univariate regression analysis was used to assess which factors were associated with LA sphericity index. Statistical analyses were performed using JMP version 14 (SAS Institute, Cary, NC, USA) and R (version 3.6.2, R Project for Statistical Computing).

## 3 RESULTS

### 3.1 Patient selection and baseline characteristics

**FIGURE 2:**
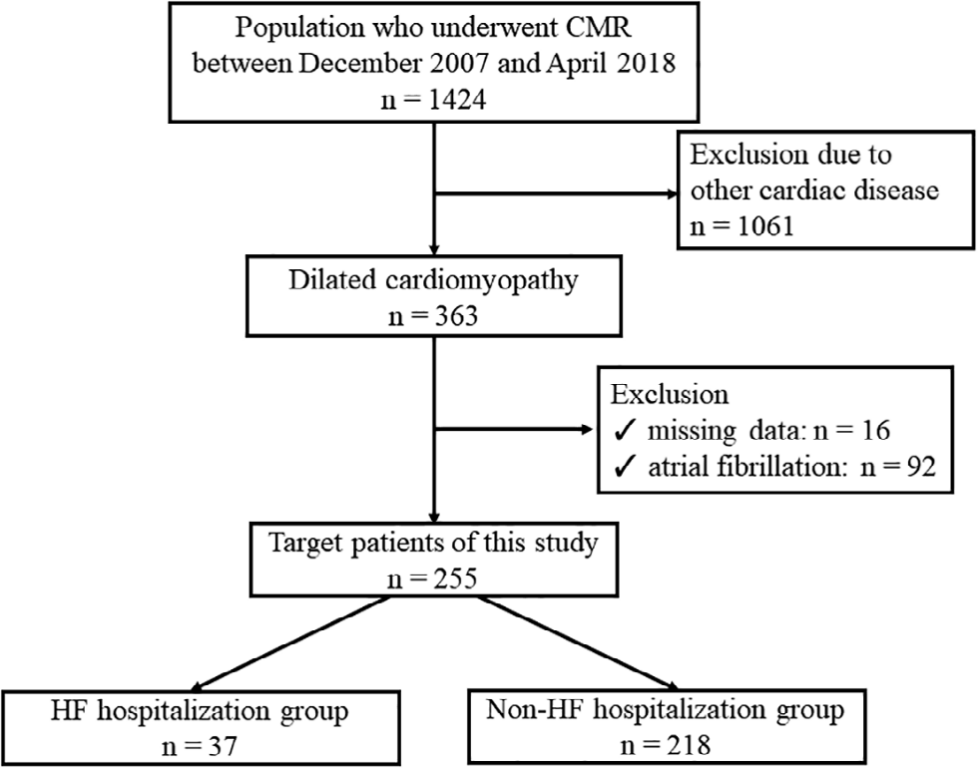
HF, heart failure

Two hundred and fifty‐five patients met the inclusion criteria for this study. During the mean follow‐up of 3.92 ± 2.89 years, 37 patients were hospitalized for HF (HF hospitalization group, Figure 2). There was no significant difference between the observation period of the HF hospitalization group and patients without hospitalization for HF (non‐HF hospitalization group; 3.07 ± 3.09 years vs. 4.06 ± 2.84 years, *p* = .056). Baseline characteristics are listed in Table 1. Age, sex, comorbidity, medications, laboratory values, and echocardiography were not significantly different between the HF hospitalization group versus the non‐HF hospitalization group. Systolic blood pressure was significantly lower in the HF hospitalization group than in the non‐HF hospitalization group.

**TABLE 1:** Patient characteristics at baseline

	HF hospitalization group (*n* = 37)	Non‐HF hospitalization group (*n* = 218)	*p* value
Age (years)	58.0 ± 16.0	55.6 ± 14.8	.354
Male, *n* (%)	28 (75.7)	164 (75.2)	.954
Systolic blood pressure (mmHg)	107.0 ± 19.0	116.6 ± 16.9	.002
Heart rate (bpm)	70.1 ± 11.5	70.6 ± 13.8	.823
Comorbidity, *n* (%)			
Diabetes mellitus	6 (16.2)	53 (24.3)	.280
Hyper tension	13 (35.1)	92 (42.2)	.419
NYHA ≥II, *n* (%)	27 (73.0)	134 (61.5)	.180
History of decompensated heart failure, *n* (%)	34 (91.9)	181 (83.0)	.170
Medications			
RASS inhibitor	37 (100.0)	205 (94.0)	.127
Beta‐blocker	35 (94.6)	207 (95.0)	.927
MRA	21 (56.8)	128 (58.7)	.823
Furosemide	28 (75.7)	137 (62.8)	.131
Laboratory values			
Serum creatinine (mg/dl)	1.0 ± 0.6	1.1 ± 1.3	.782
eGFR (ml/min/1.73m^2^)	63.6 ± 21.5	65.8 ± 20.9	.556
BNP (pg/ml)	303.4 ± 359.7	273.0 ± 367.2	.245
Echocardiography			
LAD (mm)	42.0 ± 6.7	41.9 ± 7.3	.952
LVDd (mm)	63.6 ± 8.4	61.6 ± 8.3	.193
LVDs (mm)	53.8 ± 9.7	51.8 ± 9.1	.222
LVEF (%)	31.1 ± 10.7	33.2 ± 10.8	.272
IVC (mm)	13.7 ± 3.6	14.2 ± 4.8	.575
E/A	1.3 ± 0.9	1.3 ± 0.9	.945
E/e’	15.9 ± 7.0	13.5 ± 6.2	.238
TRPG (mmHg)	23.9 ± 8.0	23.3 ± 9.9	.736
MR grade ≥ 3, *n* (%)	14 (37.8)	50 (22.9)	.053
TR grade ≥ 3, *n* (%)	1 (2.7)	20 (9.2)	.186
Cardiac magnetic resonance			
LVEDV (ml)	254.1 ± 67.8	261.0 ± 87.8	.646
LVESV (ml)	199.1 ± 68.0	194.4 ± 84.6	.751
LVEF (%)	22.7 ± 9.6	27.4 ± 10.7	.014
Cardiac index (L/min/m^2^)	2.4 ± 0.9	2.7 ± 0.9	.052
RVEDV (ml)	138.9 ± 52.5	131.3 ± 46.5	.410
RVESV (ml)	93.6 ± 46.5	85.0 ± 43.4	.313
RVEF (%)	34.2 ± 12.8	37.0 ± 14.1	.318
Presence of LGE, *n* (%)	13 (35.1)	64 (29.4)	.479
LAV_max_ (ml)	81.0 ± 30.4	75.1 ± 30.5	.282
LAV_pre‐ac_ (ml)	64.1 ± 27.8	58.7 ± 27.5	.266
LAV_min_ (ml)	48.5 ± 24.2	45.3 ± 26.7	.497
Total LAEF (%)	40.4 ± 115.4	42.6 ± 15.0	.412
Active LAEF (%)	23.7 ± 17.1	25.3 ± 16.2	.582
Passive LAEF (%)	21.7 ± 10.6	23.2 ± 10.8	.454
LA sphericity index	0.78 ± 0.35	0.58 ± 0.18	<.001

*Note*: Categorical variables are shown as numbers (percentages) and continuous variables are shown as mean ± SD.

Abbreviations: BNP, brain natriuretic peptide; eGFR, estimated glomerular filtration rate; IVC, inferior vena cava; LA, left atrial; LAD, left atrial dimension; LAEF, left atrial empty fraction; LAV, left atrial volume; LGE, late gadolinium enhancement; LVDd, left ventricular end‐diastolic dimension; LVDs, left ventricular end‐systolic dimension; LVEDV, left ventricular end‐diastolic volume; LVEF, left ventricular ejection fraction; LVESV, left ventricular end‐systolic volume; MR, mitral regurgitation; MRA, mineralocorticoid receptor antagonist; NYHA, New York Heart Association functional classification; RASS, renin‐angiotensin‐aldosterone system; RVEDV, right ventricular end‐diastolic volume; RVEF, right ventricular ejection fraction; RVESV, right ventricular end‐systolic volume; TR, tricuspid regurgitation; TRPG, transtricuspid pressure gradient.

In CMR, LV volumes and the presence of LGE were not significantly different between the two groups. LV ejection fraction was significantly lower in the HF hospitalization group than in the non‐HF hospitalization group. Although there were no significant differences in LA volume and LAEF between the two groups, the LA sphericity index was significantly higher in the HF hospitalization group than in the non‐HF hospitalization group (0.78 ± 0.35 vs. 0.58 ± 0.18, *p* < .001).

### 3.2 LA geometric parameter as a predictor of cardiac events

Univariate Cox proportional hazard analysis showed that the significant predictors of subsequent hospitalization for HF (*p* < .05) among the different clinical parameters at baseline included systolic blood pressure, LV ejection fraction, and LA sphericity index. Multivariate analysis indicated that out of these primary candidates, LA sphericity index was an independent predictor of subsequent hospitalization for HF (hazard ratio, 1.21; 95% confidence interval, 1.09–1.34, *p* < .001, Table 2).

**TABLE 2:** Univariate and multivariate analyses for the association with hospitalization for HF

	Univariate			Multivariate		
	Hazard ratio	95% CI	*p* value	Hazard ratio	95% CI	*p* value
Age	1.01	0.98–1.03	.535			
Male	1.08	0.51–2.29	.842			
Systolic blood pressure	0.97	0.95–0.99	.003	0.97	0.95–0.99	.006
Heart rate	1.00	0.98–1.03	.872			
NYHA	1.36	0.66–2.84	.396			
RASS inhibitor	<0.99		.999			
Beta‐blocker	0.87	0.21–3.63	.847			
MRA	0.99	0.52–1.92	.998			
Serum creatinine	0.99	0.59–1.23	.938			
Log BNP	1.22	0.94–1.60	.129			
E/A	1.07	0.69–1.53	.737			
E/e’	1.03	0.97–1.07	.309			
MR grade	1.50	0.76–2.95	.245			
TR grade	0.26	0.04–1.88	.093			
LVESV	1.00	0.99–1.01	.383			
LVEF	0.97	0.94–0.99	.038	0.99	0.95–1.02	.497
RVESV	0.99	0.98–1.01	.687			
RVEF	0.99	097–1.02	.610			
Presence of LGE	1.77	0.88–3.57	.120			
LAV max	1.00	0.99–1.01	.563			
Total LAEF	0.99	0.97–1.01	.428			
LA sphericity index, per 0.1 increase	1.21	1.09–1.31	<.001	1.21	1.09–1.34	<.001

Abbreviations: CI, confidence intervals; LA, left atrial; LAEF, left atrial empty fraction; LAV, left atrial volume; LGE, late gadolinium enhancement; Log BNP, logarithm brain natriuretic peptide; LVEF, left ventricular ejection fraction; LVESV, left ventricular end‐systolic volume; MR, mitral regurgitation; MRA, mineralocorticoid receptor antagonist; NYHA, New York Heart Association functional classification; RASS, renin‐angiotensin‐aldosterone system; RVEF, right ventricular ejection fraction; RVESV, right ventricular end‐systolic volume; TR, tricuspid regurgitation.

In the hard endpoint, four patients had implantation ventricular assist device and 10 patients had cardiac death during the follow‐up period. The LA sphericity index was significantly higher in patients with an implantation ventricular assist device or cardiac death than in those without (0.72 ± 0.36 vs. 0.60 ± 0.22, *p* = .044). However, univariate Cox proportional hazard analysis showed that systolic blood pressure and mitral regulation grade were the significant predictors of implantation ventricular assist device and cardiac death (*p* < .05), but LA sphericity was not. Multivariate analysis showed that systolic blood pressure was an independent predictor of implantation ventricular assist device and cardiac death (Supplementary Table S1).

**FIGURE 3:**
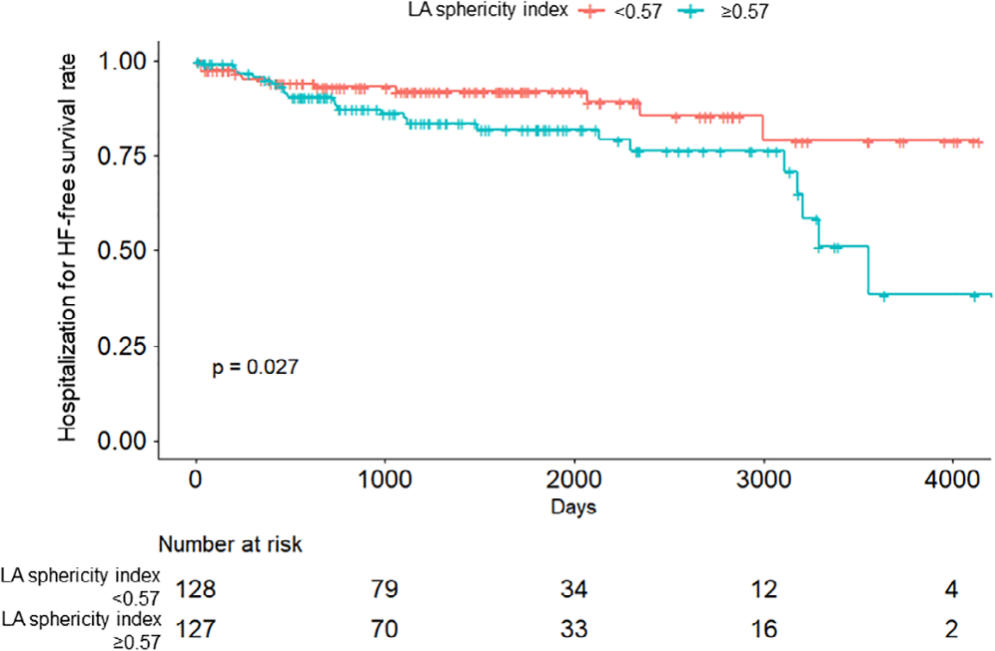
Patients were categorized based on the median of left atrial (LA) sphericity index. The Kaplan–Meier curve showed that patients with high LA sphericity index (≥0.57) had a significantly higher risk of hospitalization for heart failure (HF) than those with low LA sphericity index (<0.57)

Patients were categorized based on the median value of LA sphericity index. The Kaplan–Meier curve showed that patients with a high LA sphericity index (≥0.57) had a significantly higher risk of hospitalization for HF than those with a low LA sphericity index (<0.57, Figure 3).

Univariate regression analysis revealed that no variables were significantly associated with LA sphericity index (Supplementary Table S2); therefore, multivariate analyses were not performed.

## 4 DISCUSSION

The present study demonstrated the clinical significance of LA parameters in patients with DCM. Although there were no significant differences in LA volume and LAEF between patients with hospitalization for HF and those without, high LA sphericity index was independently associated with hospitalization for HF. LA sphericity index might be one of the important markers for prediction of HF hospitalization in patients with DCM.

### 4.1 LA geometric change

The geometric change of LA is associated with various factors such as LV function, degree of mitral regurgitation, and histological changes.^12^ The change of LA geometry into a sphere was considered to be related to the anatomical position,^19^ imbalance wall stress,^20^ histological changes in the atrial wall.^21^ Furthermore, an elevated inflammatory response, such as C‐reactive protein,^22^ interleukins, and cytokines,^23^ was shown to be involved in the geometric change of LA. In a mouse model, pressure overload was associated with LA dilatation, increased LA mass, loss of myofibrillar content, atrial cardiomyocyte hypertrophy, and atrial fibrosis.^24^ In the present study, it was considered that the geometric change of LA was caused not by specific factors but by various factors such as pressure overload, volume overload, and myocardial fibrosis.

In the present study, a high LA sphericity index, which means toward a more spherical shape, was associated with poor cardiac prognosis. The sphere transformation of LA might be caused by the stagnation of blood flow, leading to the exacerbation of HF.^25^ Moreover, it was shown that LA changes such as dilatation and failure were accompanied by LA endocrine failure and LA regulatory failure contributing to neurohumoral overactivity, vasoconstriction, and volume overload. Thus, these LA changes are thought to be associated with poor clinical outcomes.^26^

In the hard endpoint, there was no significant difference in LA sphericity index. The reason of this might be due to small number of hard outcome events and short follow period.

In the HF hospitalization group, LAV tended to be high and LAEF tended to be low. However, there were no significant differences between the HF hospitalization group and the Non‐HF hospitalization group. It could not be denied that these results were due to the small sample size. However, although the population and outcomes were different, some papers have demonstrated that LA geometric parameter had a significant difference even if there was no significant difference in LAV according to cardiac events.^27^, ^28^ Therefore, the LA geometric parameter might be a more sensitive indicator of changes in the left atrium than LAV.

Few papers have focused on LA parameters in patients with reduced ejection fraction^29^ and DCM patients.^8^ In these studies, LA volumes were measured by echocardiography. LAEF and LA geometry were not evaluated. Compared to echocardiography, CMR imaging could measure LA volume, function, and geometry more accurately. LA measurement of echocardiography sometimes underestimates LA volumes.^11^, ^12^ Therefore, we think that our method using CMR could evaluate LA function and geometry in more detail compared with the previous studies using echocardiography assessment.

A previous study showed that a high LA sphericity index was associated with the recurrence of atrial fibrillation after cardiac ablation.^16^ We have newly found that LA sphericity index was also associated with cardiac events in patients with DCM. LA sphericity index could be an essential predictive marker for cardiac events. However, there are unclear points about the mechanism by which a sphere transformation of LA leads to a poor prognosis. We think that further research is needed.

### 4.2 The usefulness of CMR

In previous meta‐analyses, the presence of LGE has been associated with poor prognosis in patients with DCM.^30^ In the present study, however, the presence of LGE was not a predictor for cardiac outcomes. A similar result has been obtained in other studies.^31^, ^32^ The reason of discordant results was unclear. The differences in cohorts, the number of events, and observation period might be related to the reasons.

LA sphericity index was calculated by a simple method using routine cine CMR without gadolinium contrast. Therefore, the LA sphericity index can provide incremental prognostic value for all patients who undergo CMR without additional cost and scan time.

### 4.3 Clinical implications

There are various parameters for predicting the prognosis of patients with DCM, and LA sphericity index could be a new parameter associated with cardiac outcome. Moreover, LA sphericity index can be measured by appending one simple analysis to routine CMR.

### 4.4 Study limitations

Several limitations need to be acknowledged in the present study. First, this was a single‐center study with a limited number of patients, which possibly resulted in a patient selection bias and lower statistical power. Second, only patients undergoing CMR were included, which way also poses a risk for bias. Third, genetic tests were not performed in our cohorts. Forth, the fact that the data were collected with the use of different devices and operators was a limitation of this study.

## 5 CONCLUSIONS

A high LA sphericity index was an independent predictor of hospitalization for HF. LA sphericity index could be one of the new parameters for predicting the prognosis in patients with DCM.

## CONFLICT OF INTEREST

The authors declare no potential conflict of interest.

## Supporting information


**Table S1** Univariate and multivariate analyses for the association with implantation ventricular assist device and cardiac death

**Table S2** Univariate analyses for the association with LA sphericity index

## Data Availability

The datasets generated and analyzed in the current study are not publicly available due to a request from our ethical committee but are available from the corresponding author on reasonable request.
