# Supplementary material for: Clinical significance of left atrial geometry in dilated cardiomyopathy patients: A cardiovascular magnetic resonance study
Source: Clin Cardiol. 2020 Dec 9;44(2):222–9. doi: 10.1002/clc.23529 (PMC7852162; doi:10.1002/clc.23529)
Supplement: Supplementary file 1 — Table S1 Univariate and multivariate analyses for the association with implantation ventricular assist device and cardiac death [file CLC-44-222-s001.docx]

**Table S1.** **Univariate and multivariate analyses for the association with implantation ventricular assist device and cardiac death**

|  | Univariate | | | Multivariate | | |
| --- | --- | --- | --- | --- | --- | --- |
|  | Hazard ratio | 95% CI | P value | Hazard ratio | 95% CI | P value |
| Age | 0.981 | 0.95-1.02 | 0.285 |  |  |  |
| Male | 1.37 | 0.38-4.92 | 0.618 |  |  |  |
| Systolic blood pressure | 0.91 | 0.87-0.95 | <0.001 | 0.92 | 0.88-0.97 | <0.001 |
| Heart rate | 0.99 | 0.95-1.03 | 0.611 |  |  |  |
| NYHA | 2.86 | 0.64-12.83 | 0.124 |  |  |  |
| RASS inhibitor | 0.54 | 0.07-4.16 | 0.588 |  |  |  |
| Beta-blocker | 0.86 | 0.11-6.59 | 0.887 |  |  |  |
| MRA | 2.85 | 0.79-10.25 | 0.080 |  |  |  |
| Serum creatinine | 0.94 | 0.24-1.41 | 0.856 |  |  |  |
| Log BNP | 1.53 | 0.99-2.47 | 0.058 |  |  |  |
| E/A | 1.34 | 0.79-2.04 | 0.253 |  |  |  |
| E/e’ | 0.98 | 0.89-1.06 | 0.631 |  |  |  |
| MR grade | 3.53 | 1.22-10.18 | 0.020 | 2.03 | 0.67-6.16 | 0.206 |
| TR grade | 0.75 | 0.10-5.74 | 0.773 |  |  |  |
| LVESV | 1.00 | 0.99-1.01 | 0.208 |  |  |  |
| LVEF | 0.96 | 0.90-1.01 | 0.107 |  |  |  |
| RVESV | 1.00 | 0.99-1.01 | 0.696 |  |  |  |
| RVEF | 0.98 | 0.94-1.02 | 0.412 |  |  |  |
| Presence of LGE | 2.61 | 0.91-7.45 | 0.079 |  |  |  |
| LAV max | 1.01 | 0.99-1.02 | 0.423 |  |  |  |
| Total LAEF | 0.97 | 0.93-1.00 | 0.064 |  |  |  |
| LA sphericity index, per 0.1 increase | 1.13 | 0.95-1.30 | 0.157 |  |  |  |

Abbreviations: CI, confidence intervals; LA, left atrial; LAEF, left atrial empty fraction; LAV, left atrial volume; LGE, late gadolinium enhancement; Log BNP, logarithm brain natriuretic peptide; LVEF, left ventricular ejection fraction; LVESV, left ventricular end-systolic volume; MR, mitral regurgitation; MRA, mineralocorticoid receptor antagonist; NYHA, New York Heart Association functional classification; RASS, renin-angiotensin-aldosterone system; RVEF, right ventricular ejection fraction; RVESV, right ventricular end-systolic volume; TR, tricuspid regurgitation.
