# Supplementary material for: Clinical significance of left atrial geometry in dilated cardiomyopathy patients: A cardiovascular magnetic resonance study
Source: Clin Cardiol. 2020 Dec 9;44(2):222–9. doi: 10.1002/clc.23529 (PMC7852162; doi:10.1002/clc.23529)
Supplement: Supplementary file 2 — Table S2 Univariate analyses for the association with LA sphericity index [file CLC-44-222-s002.docx]

**Table S2. Univariate analyses for the association with LA sphericity index**

|  | β coefficient | Univariate  95% CI | P value |
| --- | --- | --- | --- |
| Age | 0.058 | −0.001-0.003 | 0.359 |
| Systolic blood pressure | −0.023 | −0.002-0.001 | 0.715 |
| Heart rate | −0.012 | −0.002-0.002 | 0.849 |
| NYHA | 0.047 | −0.018-0.040 | 0.459 |
| Serum creatinine | −0.051 | −0.033-0.014 | 0.418 |
| Log BNP | −0.010 | −0.024-0.020 | 0.877 |
| E/A | 0.033 | −0.026-0.042 | 0.631 |
| E/e’ | 0.119 | −0.001-0.009 | 0.092 |
| MR grade | −0.006 | −0.031-0.031 | 0.925 |
| TR grade | 0.073 | −0.021-0.081 | 0.243 |
| LVESV | −0.067 | −0.001-0.0001 | 0.289 |
| LVEF | −0.017 | −0.003-0.002 | 0.782 |
| Presence of LGE | 0.016 | −0.027-0.034 | 0.805 |

Abbreviations: CI, confidence intervals; LGE, late gadolinium enhancement; Log BNP, logarithm brain natriuretic peptide; LVEF, left ventricular ejection fraction; LVESV, left ventricular end-systolic volume; MR, mitral regurgitation; NYHA, New York Heart Association functional classification; TR, tricuspid regurgitation.
